# Flanking Residues Are Central to DO11.10 T Cell Hybridoma Stimulation by Ovalbumin 323–339

**DOI:** 10.1371/journal.pone.0047585

**Published:** 2012-10-23

**Authors:** Benjamin M. Roy, Dmitriy V. Zhukov, Jennifer A. Maynard

**Affiliations:** 1 Department of Chemical Engineering, University of Minnesota, Minneapolis, Minnesota, United States of America; 2 Department of Chemical Engineering, University of Texas at Austin, Austin Texas, United States of America; Kyushu Institute of Technology, Japan

## Abstract

T cell activation requires formation of a tri-molecular interaction between a major histocompatibility complex (MHC), peptide, and T cell receptor. In a common model system, the ovalbumin epitope 323–339 binds the murine class II MHC, I-A^d^, in at least three distinct registers. The DO11.10 T cell recognizes the least stable of these, as determined by peptide-MHC dissociation rates. Using exogenous peptides and peptide insertions into a carrier protein in combination with IL-2 secretion assays, we show that the alternate registers do not competitively inhibit display of the active register four. In contrast, this weakly binding register is stabilized by the presence of *n*-terminal flanking residues active in MHC binding. The DO11.10 hybridoma is sensitive to the presence of specific wild-type residues extending to at least the P-3 peptide position. Transfer of the P-4 to P-2 flanking residues to a hen egg lysozyme epitope also presented by I-A^d^ increases the activity of that epitope substantially. These results illustrate the inherent complexity in delineating the interaction of multiple registers based on traditional thermodynamic measurements and demonstrate the potential of flanking residue modification for increasing the activity of weakly bound epitopes. The latter technique represents an alternative to substitution of anchor residues within a weakly bound register, which we show can significantly decrease the activity of the epitope to a responding T cell.

## Introduction

The adaptive immune system responds to foreign proteins through the use of cytotoxic (CD8^+^) and helper (CD4^+^) T cells that activate and direct other effectors to mount a protective response. These T cells are activated after a productive interaction between the membrane bound T cell receptor (TCR) and the surface of cells presenting a composite peptide/major histocompatibility complex (pMHC). Crystal structures have revealed regular patterns of peptide-MHC interactions [1], enabling prediction of peptide epitopes present within larger proteins. CD8^+^ T cells recognize Class I MHC molecules displaying 8–10 residue long peptides derived from intracellular proteins, proteolytically processed to a defined length in order to fit within the closed MHC binding groove. In contrast, CD4^+^ T cells recognize extracellular material that has been internalized, processed and displayed as variable length peptides in complex with heterodimeric Class II MHC molecules on professional antigen presenting cells. Because of an open-ended peptide-binding groove, Class II MHC typically display peptides with 15–20 or more residues, with the termini extending out from the binding groove [2]. These longer peptides present distinct challenges for epitope prediction since they potentiate multiple modes of interaction with the MHC [3], [4].

Class II peptides bind the MHC in an extended, polyproline II-like conformation, flanked by two antiparallel α-helices, one from each MHC chain [5]. These helices form a conserved network of hydrogen bonds with the peptide backbone spanning the length of the binding pocket that dictates a sequence independent conformation of the displayed peptide. The stability of a specific peptide is determined by side chain interactions with these helices as well as internal binding pockets. The peptide rests atop a series of pleated beta sheets, which form four prominent binding pockets, named for the respective positions within the peptide of the residues that occupy them: P1, P4, P6 and P9. The stretch of nine amino acid residues spanning these pockets is referred to as the peptide core, and the residues filling these pockets as anchor residues. Considerable effort in class II epitope prediction has focused on defining anchor residue preferences for specific MHC alleles [6]. The remaining residues within the core orient their side chains toward the helices or away from the MHC, in order to interact with the TCR.

Within the longer peptides presented by class II MHC, multiple core sequences may overlap, such that a single pMHC complex may heterogeneously present multiple cores for display to responding T cells. Murine class II I-A alleles appear particularly susceptible to this phenomenon as anchor preferences are ill-defined among the diverse set of known binding peptides. The apparent degeneracy of I-A alleles emphasizes the need for epitope prediction models that incorporate the contributions of separate registers within a single epitope. Indeed, early sequence-based prediction models found that incorporation of flanking residues outside of the predicted core improved predictions for HLA-DQ, the human homolog of I-A [7]. This appears partially attributable to flanking residues either interacting directly with MHC or influencing intracellular epitope processing [8]. More recent models have continued to incorporate flanking residues in sequence-based prediction models [4], [9], [10], with some explicitly including flanking residues for their roles in alternate register binding [3], [4].

The I-A gene is associated with numerous autoimmune mouse models, while the homologous HLA-DQ is associated with insulin dependent diabetes milletus, celiac disease, and potentially multiple sclerosis in humans (Jones et al., 2006; Kaushansky et al., 2009). This allele-specific autoimmune susceptibility may result from peptide presentation in multiple registers [11]. In this proposed mechanism, auto-reactive T cells escape thymic deletion because their target self-epitopes are hidden by “register masking,” whereby they are presented less frequently than more thermodynamically stable alternate registers within the same proteolytic fragment. Thus, T cells responding to the more stable registers are deleted, while those responding to less stable registers migrate to the periphery. At the site of inflammation, where the peptide is highly expressed, the weaker register will be presented with sufficient frequency to activate self-reactive T cells and induce disease. Specific epitopes identified in the NOD mouse model of type 1 diabetes [12] as well as experimental autoimmune encephalomyelitis [13]–[15] have been identified as potentially susceptible to this mechanism.

The DO11.10 T cell hybridoma is a popular model system used to study CD4^+^ T cell responses, originally elicited by vaccination of Balb/c mice (H-2^d^ haplotype) with hen egg white ovalbumin (OVA). The specific peptide recognized was originally described as the tryptic fragment corresponding to residues 323–339 as presented by I-A^d^ [16]. Subsequent studies first identified two distinct binding registers within this long peptide (324–332; 327–335) [17]. Later, a third register was identified (329–337) and a fourth proposed (326–334; Table 1) [18]. Among these, the DO11.10 T cell hybridoma was shown to recognize the *c*-terminal register (residues 329–337, referred to hereafter as register four) through the use of *n*- and *c*-terminal truncations and targeted amino acid substitutions [18]. The failure of MHC tetramers with a covalently linked 323–339 peptide to stain DO11.10 and other peptide-specific T cells has driven interest in further defining the register recognized. Recently, Landais et al. synthesized four tetramers, each presenting one of the four respective nine residue core sequences present within the 323–339 peptide, and confirmed that only the register four peptide stained DO11.10 cells and bound the free T cell receptor [19].

**Table 1:** Alternate registers within OVA 323–339.

Register																					Register identified *(ref)*	Dissociation rate constants^a^	
			P-4	P-3	P-2	P-1	P1	P2	P3	P4	P5	P6	P7	P8	P9							peptide used	(*k*_d_, s^−1^)
1 (324–332)						I	**S**	Q	A	**V**	H	**A**	A	H	**A**	E	**I**	N	E	A	24	323–335 Q325A^b^	0.023×10^−4^
2 (326–334)				I	S	Q	**A**	V	H	**A**	A	**H**	**A**	E	**I**	N	E	A	G	R	20	*na*	*na*
3 (327–335)			I	S	Q	A	**V**	H	A	**A**	H	**A**	E	I	**N**	E	A				19	325–336	0.026×10^−4^
4 (329–337)	I	S	Q	A	V	H	**A**	A	H	**A**	E	**I**	N	E	**A**	G	R				20	327–339 V327K^c^	6×10^−4^

a Apparent first order dissociation constants of presumed isolated registers from purified I-A^d^ for: single exponential fits to monophasic dissociation data (registers 1,3, ref 19), or back-calculated from reported half-life of dissociation (register 4, ref 23).

b Alanine was substituted at position 325 to avoid formation of demonstrated dissociation intermediate.

c Lysine was substituted at position 327 to avoid alternate register binding.

Peptide-MHC complex stability correlates broadly with immunogenicity [20], [21], as stable epitopes are more prevalent on the cell surface, and subsequently more likely to be recognized by responding T cells. Stability is commonly approximated as the dissociation rate of fluorescently labeled peptide from soluble MHC, although this approach can be complicated by highly overlapping registers. McFarland et al. showed that OVA registers one and three are long-lived and stable [17]. Supporting the thermodynamic stability of register one, a complex between the 323–339 peptide and I-A^d^ was crystallized in that register [22], and tetramers with long, unhindered covalent linkers primarily inhabit register one [19]. In contrast, the DO11.10-responsive register four peptide has a very rapid off-rate, with a half-life of around twenty minutes [21]. To minimize alternate register effects confounding this measurement, the amino acid sequence flanking the register four core was truncated at P-2 on the *n*-terminus, with the P-2 position altered from valine to lysine, which is presumably too large to stably occupy the P1 binding pocket. These peptide dissociation rates predict that register four would behave as a masked, cryptic epitope, albeit in the context of a foreign antigen. Despite this kinetic data, seemingly contradictory accounts have emerged as to whether register four is masked *in vivo* by its overlapping counterparts. Two reports observed that register four specifically elicits relatively few responding T cells [21], [23]. In contrast, Landais et al. found that pMHC tetramers presenting only the fourth register stained a significantly greater number of responsive T cells following OVA immunization than tetramers presenting the more stable first and third registers [19].

Here, we set out to quantify the extent to which registers one and three mask display of the active register four, using synthetic OVA peptides incorporating truncations and/or amino acid substitutions to alter presentation of a given register and, ultimately, to enhance register four display. Recombinant proteins harboring internal peptide variants were employed to capture effects associated with epitope processing. After incubation of DO11.10 hybridomas with corresponding B-cells and a peptide variant, we measured T cell activation in terms of IL-2 secretion, as the most sensitive determinant of register four’s accessibility to responsive T cells.

We observed that presentation of register one, but not register three, is independent of register four activity. Disruption of register one through *n*-terminal peptide truncation had no impact on measured activity. Residues thought to be primarily involved with register three binding, on the other hand, proved essential for activity. Wild type residues 325–327 (QAV; P-4 through P-2) were necessary for complete activation. Transfer of these flanking residues to identical positions on an unrelated hen egg white lysozyme epitope, HEL11–25, increased the activity of that epitope dramatically, based on EC_50_ and maximal response. This suggests that these residues independently stabilize peptide-I-A^d^ interactions and that, consequently, register four stability has been underestimated by techniques truncating these flanking residues. Attempts to determine the precise effect of the adjacent register three stability on activity of the weaker register four using anchor substitutions were inconclusive. Collectively, our results indicate that flanking residues can affect activity to a previously unforeseen extent. These results have broad implications for class II epitope prediction algorithms and protein engineering efforts to modulate protein immunogenicity in therapeutics and vaccines. In particular, the engineering of flanking residues may increase the stability of weakly bound epitopes for use in vaccination strategies and the manipulation of immunogenicity within proteins.

## Results

### DO11.10 Activation is Modulated by Epitope Anchor Residues Substitutions

The rapid dissociation of register four relative to the other registers within 323–339 led us attempt to increase peptide activity through a corresponding increase in register four affinity for I-A^d^. The poor stability of register four is likely due to the weakly binding anchor residues at ovalbumin positions A329, A332, I334, A337 (Table 1). Anchor substitution is a common technique for increasing the affinity of a given register; previously, Chaves et al. reported the relative stability of commonly observed anchor residues with I-A^d^ [24]. In this and other work, the interactions of each anchor residues with its respective binding pocket were found to be independent of the overall sequence [3].

**Figure 1:**
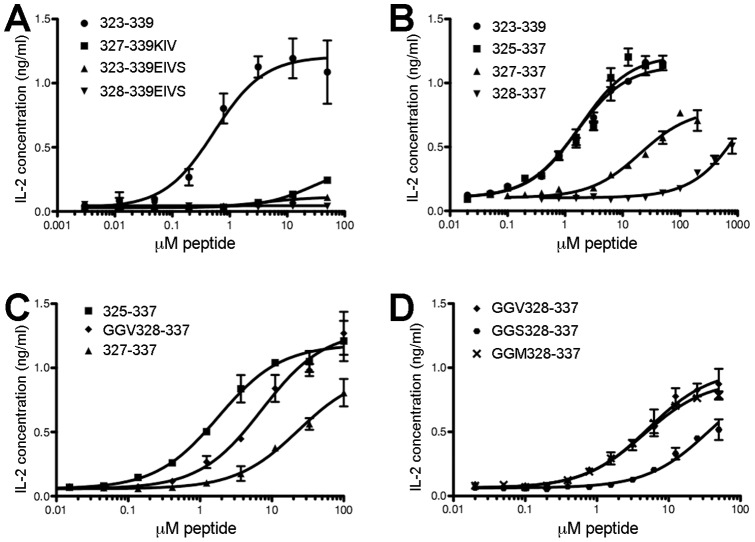
Specific flanking residues but not alternate register stabilization enhances OVA peptide activity. DO11.10 hybridoma and antigen presenting cells were incubated overnight in the presence of exogenous peptide variants, followed by quantification of IL-2 released as a measure of T cell activation. ***A*** **,** Wild-type ovalbumin 323–339 (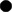) was compared with peptide variants modified at anchor residues P4 (A to I) and P6 (I to V) in conjunction with additional changes in an effort to stabilize the active register four. Peptide variants include 327-339KIV (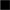) with an additional *n*-terminal truncation and P-2 modification (V to K) to prevent register shifting; 323-339EIVS (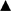) the full-length peptide with all four anchor residues altered to strongly binding residues; and 328-339EIVS, an *n*-terminal truncation of the previous variant (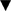). The cell culture media contained 2 mM glutamine for this assay. ***B*** **,** In a subsequent effort to stabilize register four by removing alternate register anchors, the wild-type 323–339 peptide (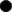) was compared with *n*-terminally truncated variants 325–337 (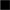), 327–337 (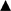), and 328–337 (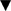). ***C*** **,** To determine whether peptide length is responsible for the activity differences observed in the truncated variants, the 325–337 variant (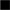) which includes register four and the *n*-terminal P-1 to P-4 residues was compared with GGV328–337, in which the P-3 and P-4 positions were replaced with glycine (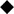) and a further truncated variant, 327–337 (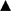), lacking the P-3 and P-4 residues. ***D*** **,** To determine whether binding of register three enhances register four activity, we changed the register three P1 anchor residue (also register four P-2) to amino acids predicted to stabilize (M) or destabilize (S) register three. We compared three variants with identical length: variant GGV328–337 (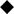) with a wild-type V versus GGS328–337 (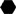) and GGM328–337 (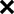). Data were fit to three-parameter curves with Graphpad Prism and are representative of three independent experiments; error bars show standard deviation of triplicate wells assayed for a given peptide concentration.

Two previous reports described anchor substitutions to stabilize register four. Robertson et al. replaced all four positions with the corresponding anchor residues from a sperm whale myoglobin epitope possessing high affinity for I-A^d^ as follows: A329E, A332I, I334V, A337S (the resulting peptide is referred to here as 323–339 EIVS) [18], and reported increased activity for the variant. Similarly, Lazarski et al. reported hybridoma recognition of a variant incorporating two of these substitutions, A332I and I334V (here called 327–339 KIV), as well as a V327K substitution intended to prevent register shifting to the more stable third register via incompatibility of the bulky lysine with the P1 pocket [21]. To assess the relative success of these attempts, we synthesized both variants, along with an *n*-terminally truncated version of the Robertson peptide (328–339 EIVS; Table 2) to remove the effects of flanking residues which we hypothesized would promote register masking. To assess peptide activity, DO11.10 hybridomas were incubated for 24 hours with I-A^d+^ A20 lymphoma cells in the presence of peptide, and supernatant was subsequently assessed for the presence IL-2 by a matched pair ELISA as a measure of T cell activation. Surprisingly, all three peptide variants drastically reduced T cell stimulation versus 323–339 (Figure 1A). Given the documented affinity increase of 327–339 KIV for I-A^d^ [21], the decrease in T cell stimulation associated with these variants likely results from interference in TCR/pMHC recognition due to minor structural variations.

**Table 2:** Sequences and activities of OVA peptide variants.

Peptide			P-4	P-3	P-2	P-1	P1	P2	P3	P4	5P	P6	P7	P8	P9			EC_50_ (µM peptide)	95% Confidence	Max response (ng/ml IL-2)	95% Confidence
*OVA #*	323	324	325	326	327	328	329	330	331	332	333	334	335	336	337	338	339				
323–339	I	S	Q	A	V	H	**A**	A	H	**A**	E	**I**	N	E	**A**	G	R	1.6	1.3–2.0	1.1	1.0–1.2
325–337			Q	A	V	H	**A**	A	H	**A**	E	**I**	N	E	**A**			1.7	1.2–2.3	1.1	0.9–1.2
327–337					V	H	**A**	A	H	**A**	E	**I**	N	E	**A**			12	7–23	0.7	0.5–0.9
328–337						H	**A**	A	H	**A**	E	**I**	N	E	**A**			ND		nd	
GGV328-337			G	G	V	H	**A**	A	H	**A**	E	**I**	N	E	**A**			6	4–10	1.1	0.9–1.4
GGS328-337			G	G	S	H	**A**	A	H	**A**	E	**I**	N	E	**A**			12	7–20	1.0	0.7–1.3
GGM328-337			G	G	M	H	**A**	A	H	**A**	E	**I**	N	E	**A**			6	4–9	1.0	0.8–1.2
327-339 KIV					K	H	**A**	A	H	**I**	E	**V**	N	E	**A**	G	R	ND		nd	
323-339 EIVS	I	S	Q	A	V	H	**E**	A	H	**I**	E	**V**	N	E	**S**	G	R	ND		nd	
328-339 EIVS						H	**E**	A	H	**I**	E	**V**	N	E	**S**	G	R	ND		nd	

EC_50_ and maximum response values from 3-parameter curve fits of aggregate data from multiple (at least two) experiments. Positional numbering is in relation to the active register four.

### DO11.10 Stimulation Modulated by Residues *n*-terminal to P-2

The failure of anchor four residue substitutions to increase activity led us to explore *n*-terminal truncations alone as a means to destabilize alternate registers, which may compete with register four for MHC binding. The 323–339 peptide was progressively truncated from the *n*-terminus, with each truncated peptide extending to an amidated residue 337 and each peptide variant incubated with DO11.10 and A20 cells, as above. The 325–337 peptide, lacking the P-1 and P1 positions of register one, conferred identical stimulation in comparison to the 323–339 peptide, in terms of the 50% effective concentration (EC_50_) and maximal response, suggesting that register one does not effectively compete with register four for display (Figure 1B, Table 2).

Further truncations intended to destabilize register three reduced activity dramatically. The 327–337 peptide lacks positions P-3 and P-4 relative to the active register, which would seem too distant to be relevant, yet the EC_50_ increases nearly 10-fold, from 1.6 to 12 µM peptide while the maximal response decreases from 1.1 to 0.7 ng/mL IL-2. For the 328–337 peptide, further lacking position P-2, activity is so severely reduced that accurate EC_50_ values could not be determined (Table 2). These data agree qualitatively with Robertson et al. who measured T cell proliferation from transgenic DO11.10 mice in response to a similar set of truncated peptides [18]. DO11.10 stimulation is thus found to be maximal when in addition to active register four, the presumably competing third register (residues 327–335) is completely present along with at least one additional *n*-terminal residue.

The necessity of these distant flanking residues lends itself to three possible explanations. (1) The necessity of these flanking residues is a result of their direct interaction with the responding T cell. (2) Despite their distance from the active register, these residues stabilize it by means of non-traditional interaction with the MHC. (3) The primary role of these flanking residues is to stabilize register three, which on the whole cooperatively promotes, rather than competitively inhibits, display of register four.

### DO11.10 Flanking Residues Increase Activity upon Transfer to HEL1125 Peptide

In order to test these competing hypotheses, we employed an unrelated peptide epitope from the hen egg white lysozyme protein (HEL), which is presented by the same MHC (I-A^d^) as the ovalbumin peptide to a distinct responding T cell [25]. A survey of the available pMHC crystal structures reveals that the T cell receptor can, by virtue of its CDR1α and CDR3α loops, contact presented flanking residues in the P-1 position and occasionally at more distant flanking positions [1]. MHC contacts, on the other hand, appear to be more predictable, and are typically limited to canonical hydrogen bonds between residue P-1 and the side chain of β81H or Y, as well as residue P-2 and the main chain of α53 [26].

The HEL epitope consisting of residues 11 through 25 (HEL11-25) has been characterized as a weak MHC binder when presented by I-A^d^, with a short half-life of approximately six hours [21] that presumably results from a poorly accommodated arginine side chain in the P1 pocket. We compared the activities of wild type HEL11-25 to a variant in which the native P-4 to P-2 sequence was replaced with OVA-specific residues (QAV13-25) and a control in which these three residues were adjusted to GGS (GGS13-25). Since these changes lengthen the HEL11-25 peptide by a single amino acid, a control in which the HEL11-25 peptide was appended with an *n*-terminal glycine in position P-3 (G11-25) was also analyzed. We reasoned that if the variant including the OVA flanking residues (QAV13-25) showed increased activity, it would increase the likelihood that these flanking residues are interacting with the presenting MHC, rather than the distinct responding TCR.

**Figure 2:**
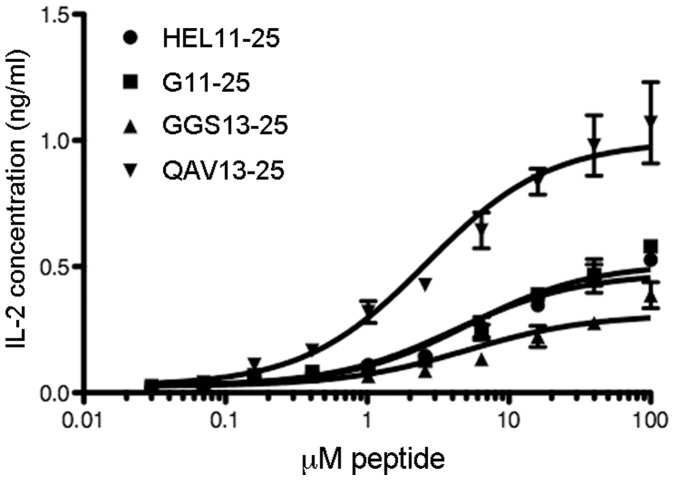
Ovalbumin flanking residues enhance HEL11-25 peptide activity. HEL11-25 hybridoma cells and antigen presenting cells were incubated overnight in the presence of exogenous peptide variants, followed by quantification of IL-2 release as a measure of T cell activation. The wild type HEL11-25 peptide (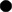) is compared to a longer variant with an additional G at position P-4, G11-25 (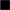); an additional control variant in which the P-2 to P-4 flanking residues have been replaced with GGS, GGS13-25 (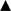); and a variant presenting the ovalbumin- flanking residues in positions P-4 to P-2, QAV13-25 (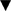). Data were fit to three-parameter curves with Graphpad Prism and are representative of three independent experiments; error bars show standard deviation of triplicate wells assayed for a given peptide concentration.

Interestingly, the QAV13-25 variant significantly increases HEL peptide activity, with the EC_50_ decreasing two-fold, from 6 to 3 µM peptide, while the maximum response increases two-fold, from 0.7 to 1.5 ng/mL IL-2. In contrast, the control peptides exhibit minimal or no change in activity, as measured by EC_50_ and maximal response (Figure 2; Table 3). The OVA flanking residues at positions P-4 through P-2 independently increase peptide activity in a manner independent of the specific T cell receptors involved, which does not support hypothesis one above. It does, however, support hypothesis two and a direct role for the QAV *n*-terminal flanking residues in stabilizing the peptide-MHC interaction. In addition, HEL11-25 has not been shown to occupy multiple frames in I-A^d^, thus reducing the possibility that flanking residues are relevant in any role apart from stabilization of the active register (hypothesis three). Collectively, these data support hypothesis two over the proposed alternatives.

**Table 3:** Sequences and activities of HEL peptide variants.

Peptide						11		12		13		14		15		16		17		18		19		20		21		22		23		24		25		EC_50_ (µM peptide)		95% Confidence		Max response (ng/ml IL-2)		95% Confidence	
HEL11-25						A		M		K		**R**		H		G		**L**		D		**N**		Y		R		**G**		Y		S		L		6		2–20		0.7		0.3–1.1	
G11-25				G		A		M		K		**R**		H		G		**L**		D		**N**		Y		R		**G**		Y		S		L		7		2–23		0.7		0.3–1.1	
QAV13-25				Q		A		V		K		**R**		H		G		**L**		D		**N**		Y		R		**G**		Y		S		L		3*		1–6		1.5*		0.9–2.1	
GGS13-25			G		G		S		K		**R**		H		G		**L**		D		**N**		Y		R		**G**		Y		S		L		6*		2–26		0.5*		0.2–0.8		

EC_50_ and maximum response values from 3-parameter curve fits of aggregate data from multiple (at least two) experiments.

* p<0.01 by extra sum of squares f-test of curve fits for antigen vs. control (HEL11–25 for HEL peptides). Comparisons performed only on data from assays in which both antigens were tested (minimum of two independent assays).

### Peptide Activity is Specific to Wild Type Residues *n*-terminal to P-2

To further clarify the role of residues 325 and 326 in the register four interaction with I-A^d^, we examined the necessity of wild type identities in these positions. Published crystal structures show conserved hydrogen bonds between main chain amino acids of presented peptides at positions P-1, with the side chain of histidine or tyrosine at β81 of the MHC, and P-2, with the main chain of α53. Observed hydrogen bonds and van der Waals interactions between MHC and more distant *n*-terminal flanking positions are irregular and rare.

We replaced the Q325 and A326 residues with glycine in a synthetic peptide (GGV328-337). Glycine was selected over alanine because of the latter’s propensity to occupy I-A^d^ anchor positions, as well as the presence of alanine at position 326 in the native epitope. The resulting variant GGV328–337 was more active than the shorter 327-337 peptide (EC_50_ = 6 and 12 µM, respectively), but remained three-fold less potent than 325-337 (EC_50_ = 2 µM; Figure 1C, Table 2), both differences are statistically significant (p<0.01). For DO11.10, peptide activity is somewhat dependent on wild-type residues extending at the *n*-terminus to at least the register four P-3 position (residue 326).

### Carrier Proteins Presenting Variant Peptides Recapitulate Synthetic Peptide Results

To confirm the peptide results and explore their relevance in the context of the native Class II peptide display process, we introduced select peptide variants into a carrier protein, maltose binding protein (*malE*). If flanking residues are involved in register four stabilization through MHC interaction, then their presence should have the same effect in fusion proteins as in exogenous peptides. However, processing and subsequent presentation has been reported to eliminate display of weaker pMHC conformers observed during exogenous peptide display via the MHC Class II-like endosomal protein H2-DM [25], [27]–[30]. We wondered whether the presentation of register four and stabilization via distant flanking residues was the result of a conformer uniquely achieved in the context of exogenous peptides, one that might be hindered by more stable competing registers when subjected to endosomal MHC loading conditions.

**Figure 3:**
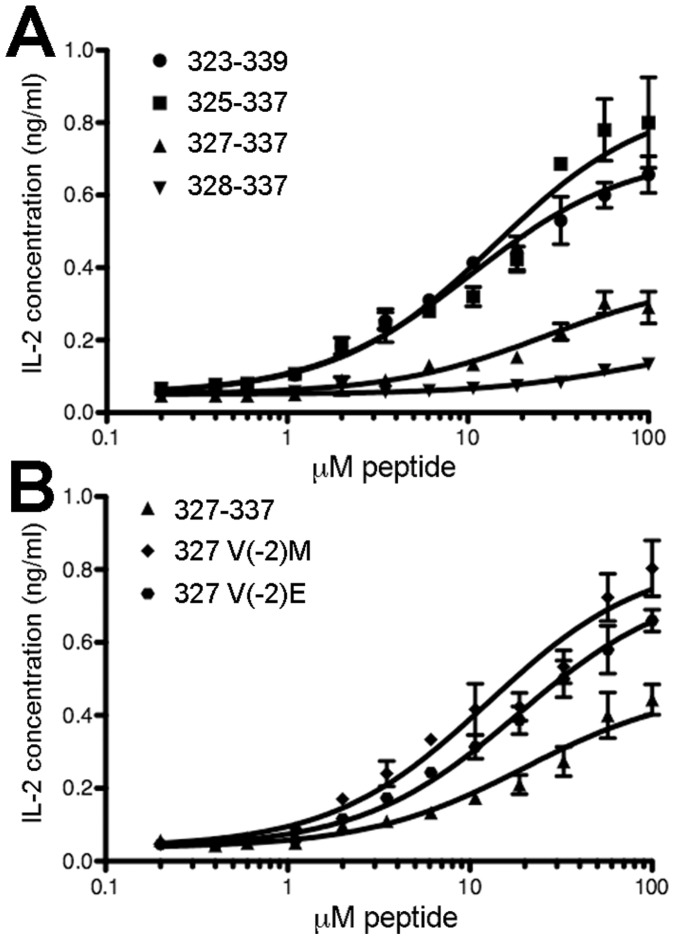
Specific flanking residues and register three enhance Ova peptide activity when endogenously processed as *malE* chimeras. DO11.10 hybridoma and A20 antigen presenting cells were incubated overnight in the presence of *malE* chimeras containing ovalbumin epitope variants, followed by quantification of IL-2 release as a measure of T cell activation. *A*, Comparison of wild-type peptide 323–337 insert (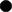) with *n*-terminally truncated variants lacking alternate register anchor positions: 325–337 (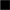), 327–337 (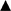), and 328–337 (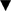). ***B,*** A truncated control peptide including the entire register four and register three, 327–337 (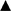) was compared with variants in which the register three P1 anchor was altered in order to increase this registers stability: 327 V(-2)M (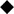) and 327 V(-2)E (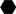). Data were fit to three-parameter curves with Graphpad Prism and are representative of two independent experiments; error bars show standard deviation of triplicate wells assayed for a given protein concentration.

Peptide sequences were introduced into the exposed loop in place of residues 133–140 as first described by Martineau *et al.* [31], with a glycine-serine extension (GSGSG) appended to the *n*-terminus of inserted epitopes to increase solubility (Figure 3A, Table 4). The activity of the *n*-terminally truncated peptides in MBP (325–337, 327–337, 328–337) indicates a maintained preference for wild type flanking residues as was previously observed with synthetic peptides (Figure 3A, Table 4). The wild-type 323–337 and 325–337 inserts have EC_50_ within error (13 µM with a max signal of 0.8 ng/mL and 18 µM protein with a max signal of 1.1 ng/mL, respectively) while the activities of the 327–337 and 328–337 variants are greatly reduced (EC_50_ of 41 and 73 µM, with max signals of 0.6 and 0.3 ng/mL, respectively). The increased activity of variants retaining the wild type P-3 and P-4 residues of register four during intracellular processing again supports their direct role in mediating the peptide and MHC interaction.

**Table 4:** Sequences and activities of peptide variants in MalE carrier protein.

MalE chimera	323	324	325	326	327	328	329	330	331	332	333	334	335	336	337	338	339		EC_50_ (µM peptide)	95% Confidence	Max response (ng/ml IL-2)	95% Confidence
323–337	I	S	Q	A	V	H	**A**	A	H	**A**	E	**I**	N	E	**A**				13*	8–20	0.8*	0.6–1.0
325–327			Q	A	V	H	**A**	A	H	**A**	E	**I**	N	E	**A**				18*	12–25	1.1*	0.9–1.4
328–337						H	**A**	A	H	**A**	E	**I**	N	E	**A**				73*	26–201	0.3*	0.1–0.5
327–337					V	H	**A**	A	H	**A**	E	**I**	N	E	**A**				41	27–61	0.6	0.5–0.8
327 V(-2)M					M	H	**A**	A	H	**A**	E	**I**	N	E	**A**				18*	13–25	1.0*	0.8–1.2
327 V(-2)E					E	H	**A**	A	H	**A**	E	**I**	N	E	**A**				25*	15–44	0.7*	0.5–1.0

EC_50_ and maximum response values from 3-parameter curve fits of aggregate data from multiple (at least two) experiments. Positional numbering is in relation to the active register (register four in the case of OVA).

* p<0.01 by extra sum of squares f-test of curve fits for antigen vs. control (MalE 327–337 for MalE chimeras). Comparisons performed only on data from assays in which both antigens were tested (minimum of two independent assays).

### Substitutions at Position 327 Enhance Peptide Activity within a Carrier Protein

Register four stabilization by distant flanking residues, shown above, does not preclude the involvement of register three in cooperatively increasing the presentation of the active register (hypothesis three), or inhibiting it, for that matter. After all, residues 325–327 would, according to traditionally understood peptide-MHC interactions, be more likely to act as P-2 through P1 of register three than as P-4 through P-2 of register four. We thus sought to specifically probe the impact of changes in the relative stability of register three on the activity of register four.

Initially, we introduced substitutions for valine at position 327 to modulate register three stability. Among the candidate register three anchor residues (V327, A330, A332, N335), the P1 position occupied by V327 is the only anchor not overlapping with the register four core (329–337). Previously, residue substitutions in the spacious first binding pocket of I-A^d^ have been shown to have drastic impacts on register stability [24]. For the carried peptide, two variants of the *malE* 327 were created with strong predicted P1 anchors for register three: 327 V(-2)M and 327 V(-2)E. The negatively charged glutamic acid side chain contrasts with the nonpolar methionine in potential contributions to solubility and protease accessibility, but both are strongly preferred in the P1 binding pocket [24]. Both protein variants enhanced activity of the processed epitope about two-fold, based on EC_50_ values (18 and 25 µM for the variants versus 41 µM for the native 327–337 fusion protein; Figure 3B), possibly indicating a positive relationship between register three stabilization and register four activity.

### Increased Activity of Position 327 Mutants is not Dependent on H2-DM

**Figure 4:**
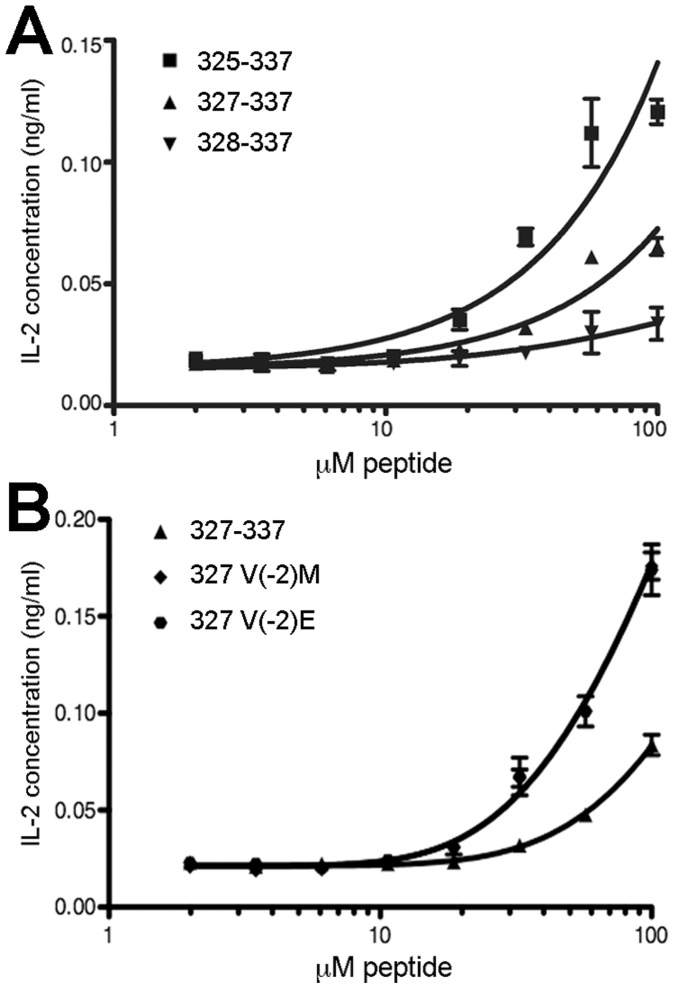
H2-DMα is not responsible for the different activities of endogenously processed peptide variants. DO11.10 hybridoma and 3A5 antigen presenting cells containing mutant H2-DMα gene, were incubated overnight in the presence of *malE* chimeras containing ovalbumin epitope variants, followed by quantification of IL-2 release as a measure of T cell activation. ***A*** **,** To assess the effects of removing alternate register anchor positions, truncated ovalbumin peptide inserts 325–337 (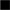), 327–337 (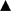), and 328–337 (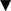) were compared. ***B*** **,** To assess the effect of stabilizing register three with P1 anchor substitutions, the control peptide 327–337 (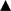) was compared with variants 327 V(−2)M (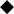) and 327 V(−2)E (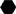), substitutions which have been shown to confer enhanced I-A^d^ binding. Curve fits for the latter two are nearly identical, and lie atop one another. Data were fit to three-parameter curves with Graphpad Prism and are representative of two independent experiments; error bars show standard deviation of triplicate wells assayed for a given protein concentration.

During peptide loading onto the MHC in the endosomal compartment, the class II-like H2-DM protein modulates the repertoire of displayed peptides [28], [30]. To examine whether the activity of our chimeras depends on H2-DM activity, we substituted 3A5, a variant of the A20 cell line with an inactivated H2-DMα chain [34], [35] in our stimulation assays. While these experiments were less sensitive, the relative activities of the peptide variants were consistent with A20 experiments for the peptide length controls (Figure 4A). Likewise, the enhanced activity of methionine and glutamic acid substitutions at position 327 was maintained during processing by these cells (Figure 4B).

Overall, the use of the *malE* carrier protein to mediate peptide presentation supports the role of flanking residues in stabilizing register four presentation, with sequence preferences maintained upon intracellular processing in a H2-DM-independent manner. This supports a role for flanking residues in interacting with the MHC, either through stabilizing the recognized conformer or an intermediate binding state.

### Increased Activity of Position 327 Mutants is not Replicated in Free Peptides

To confirm the relationship between register three stability and register four activity seen in position 327 variants in chimeras above, we made similar changes in the free peptide. Two variants of GGV328–337 were created with substitutions at the 327 position (P1 for register three): one in which the valine was replaced with a methionine (GGM328–337), and a second in which the valine was replaced with serine (GGS328–337), which can act as an anchor, but poorly occupies P1 [22]. If register four is cooperatively aided by register three binding, we would expect peptide activity to correlate with P1 anchor preference, such that GGM > GGV > GGS. In the case of the free peptides GGS328–337 shows two-fold reduced EC_50_, consistent with this weak relationship. However, GGM328–337 is nearly identical in activity to GGV328–337 (Figure 1D; Table 2).

Presentation of register four may be limited by the more stringent competition for MHC binding between a variety of epitopes during endogenous peptide loading. In general, greater concentrations of the carrier protein are needed to achieve the same stimulation as with exogenous peptides (Table 4). In addition to the invariant chain peptide, which shows relatively high affinity for I-A^d^ [32], *malE* itself contains several immunogenic determinants with which the inserted epitope must compete [33].

However, the failure of GGM328–337 to improve activity may indicate that substitutions at position 327 have an effect distinct from increasing register three stability. Direct measurement of peptide dissociation rates between I-A^d^ and P1 variants derived from *malE* fusion proteins from is complicated by the same overlapping registers inherent in the wild type peptide. In the absence of reliable kinetic data, it is impossible to know for sure whether predicted changes in register three stability as a result of position 327 substitutions were actually realized. The absence of strict agreement between predicted changes in register three stability and peptide activity somewhat weakens the case for cooperativity between the two registers.

### Molecular Docking Predicts Registers 1 and 3 Interactions with MHC

To further probe the connections between binding registers and flanking residue interactions with the MHC, we used the molecular docking program ClusPro in conjunction with the experimental I-A^d^ structure to predict peptide-MHC interactions [36]. Subsequent docking of the OVA peptide yielded high scoring structures with the peptide oriented in register one as seen in the crystal structure, and register three, but neither registers two nor four (Supplementary Figure 1).

## Discussion

Of the four registers present within the ovalbumin 323–339 peptide, the first register (residues 324–332) forms the most kinetically stable complex with MHC [17], is preferentially presented in I-A^d^ tetramers [19], and has been crystallized [22]. When *n*-terminally truncated to position 325, the peptide can no longer occupy this first register due to the absence of the register one P1 anchor and P-1 flanking residue [17]. Yet in the presence of the responsive DO11.10 hybridoma, 325–337 is no more active than the full-length version, indicating that register one does not competitively inhibit display of the active register four. In contrast, the presence of an intact register three enhances peptide activity relative to the isolated register four: the activity of the short 328–337 peptide is lower than that of the 327–337 peptide, which is in turn lower than the 325–337 peptide.

**Figure 5:**
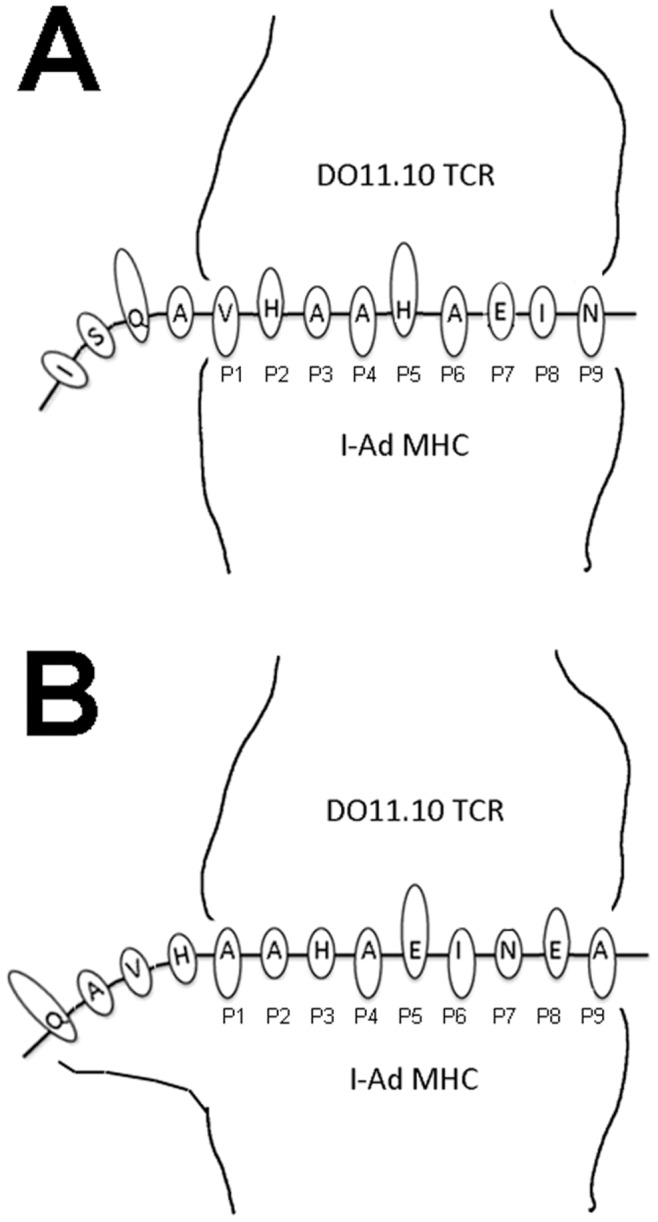
Cartoons depicting the tripartite interactions between the DO11.10 TCR, I-A^d^ MHC and the OVA 323–339 peptide in various registers. ***A***, The peptide is shown in register three, with the MHC anchor residues (P1, P4, P6 and P9) extending towards the MHC and residues interacting with the TCR (primarily P5 and to a lesser extent, P2 and P8) extending towards the TCR. ***B*** *,* The peptide is shown in register four, with MHC anchor residues and TCR residues shown as above. The cartoon MHC has been modified to indicate potential interaction with flanking residues extending as far as P-4.

Our efforts to dissect OVA 323–339 interactions with MHC led us to conclude that register four presentation depends upon distant flanking residues interacting with I-A^d^ (Figure 5). This is based on the observations that wild type residues extending to at least P-3 are necessary for maximum activity, and the interaction of flanking residues with the presenting MHC generalize to the HEL11–25 peptide. Substitution of P-4 and P-2 residues into the distinct I-A^d^ epitope, HEL11–25, dramatically increases peptide activity when presented to a responsive T cell hybridoma. Within the available peptide/MHC crystal structures, flanking residues form hydrogen bonds and van der Waals contacts with the MHC, commonly with P-1 and P-2, but interactions at P-3 and beyond are not unprecedented [39]–[45].

The common practice of truncating or substituting flanking residues may explain why register four has been reported to be both ineffective at producing T cell responders [21], [23], and immunodominant [19]. These modifications may have also resulted in artifactually weak estimates of peptide dissociation rates. These measurements used a minimal register four peptide truncated at position 327, with 327 itself altered from valine to lysine (327–339 KIV) [21] to eliminate register three effects. Since these changes also remove or alter P-4 to P-2 flanking residues we have shown to be critical for activity, use of this minimal peptide likely results in a much faster off-rate than is relevant for the larger 323–339 peptide.

Despite the observation that distant flanking residues are important for register four presentation, this does not resolve all questions surrounding the epitope. Although we suspect that the stability of the active register has been previously underestimated, this is probably insufficient to eliminate competition from the stable registers one and three. Why then, do we not observe masking of the register four in presentation to the hybridoma?

Register masking has attracted increasing attention in recent years as a mechanism for promoting autoimmunity via incomplete thymic education. For instance, the weakly bound myelin basic protein epitope (MBP ^Ac^1–9) is flanked at the *c*-terminus by a more stable register [13], [15], as well as at the *n*-terminus in the golli-form of MBP expressed in the thymus [14]. Maverakis et al. observed direct competition between registers for MHC binding, as presentation of each register was reduced by the presence of an alternate register, as measured by T cell stimulation. In the non-obese diabetic mouse model of type 1 diabetes, the insulin B 9–23 peptide was also found to bind in multiple registers [37], [38], with the least stable register apparently activating pathogenic T cells [12]. Understanding the distinction between these epitopes and the apparently unmasked register four of OVA 323–339 is likely prove insightful in the future investigation of autoimmunity.

In seeking to understand the lack of register masking in 323–339, we attempted to directly manipulate the stability of register three through amino acid substitutions and observe its effect on register four activity as measured by hybridoma stimulation. Methionine and glutamic acid substitutions at position 327 predicted to stabilize register three actually increased the activity of register four when processed from a *malE* carrier protein and presented by A20 cells. This raises the intriguing possibility that register four display is cooperatively enhanced by a stable register three. However, methionine substitution at 327 in the context of an exogenous peptide did not increase activity. Given our uncertainty at whether substitutions actually produced the desired register three stabilization, and the difficulty of measuring this stabilization in the face of entangling register kinetics, we cannot claim with sufficient confidence that such a cooperative effect exists.

As a final note, our finding that the anchor substituted peptide 323–339 EIVS severely diminished activity contrasts with the report of Robertson *et al.* [18]. Many reports have described successful increases in biological activity via anchor swaps, particularly for CD8^+^ T Cells [47]–[53]. However, the effect seems far from general, as several other reports have shown the opposite effect include [43], [54], [55]. Notably, our work is consistent with Landais et al., who generated I-A^d^ tetramers with the register four 329–337 peptide and observed reduced DO11.10 staining when the chemically distinct P1 substitutions A329M or A329E were introduced [19]. The discrepancy with Robertson et al. may be attributable to assay differences: measurement of hybridoma IL-2 secretion versus their DO11.10 transgenic T cell proliferation. In summary, this result highlights the sensitivity of the DO11.10 TCR to even small conformational changes introduced within the core. Inconsistency in anchor residue substitution effects among different peptides makes the prospect of stabilization through substitution of flanking residues all the more enticing.

### Conclusions

Overall, our findings support inclusion of residues outside the nonameric core and multiple, distinct registers in epitope prediction models [3], [4], [9], [10]. The ability of the ovalbumin flanking residues to confer enhanced activity to an unrelated HEL11–25 peptide may have general applications for understanding peptide immunogenicity and stability. In terms of the specific ovalbumin 323–339 peptide, our attempts to increase peptide activity through register four anchor substitutions severely diminished activity. Anchor substituted peptides may be more prone to influencing T cell response, as compared to flanking residue changes which are more distal to primary T cell contacts. Modification of flanking residues may increase activity of a weakly bound peptide while minimizing changes in the responding T cell repertoire. Recently described high throughput techniques [56], are likely to be instrumental in identifying stabilized variants of weakly bound peptides for use as altered peptide ligands in autoimmune therapies and vaccines.

## Materials and Methods

### OVA and HEL Peptides

Synthetic ovalbumin 323–339 peptide (p323) was purchased from Anaspec (#62571, Fremont, CA). Custom ovalbumin (OVA) and hen egg lysozyme (HEL) peptide variants were synthesized by Peptide 2.0 (Tables 2 and 3, Chantilly, VA). Peptides are named based on the location of the sequence in the linear protein, with amino acid residues substitutions indicated. For instance, a peptide representing residues 328–337 from ovalbumin is termed OVA328–337, while the same peptide with three extra flanking residues (two glycines and a valine) on the n-terminus is termed GGV328–337 and a slightly shorter peptide with three internal anchor residues substitutions (lysine, isoleucine and valine) is 329-337KIV. All ovalbumin variants were amidated at the c-terminus. All peptides were 95% pure or greater as measured by HPLC analysis, with multiple peptide lots used to confirm results.

### Chimeric *malE* Proteins Containing OVA Epitopes

Fragments of ovalbumin were introduced into the maltose binding protein (*malE*) in a manner similar to that first described by Martineau et al. (1992). These short peptide sequences were introduced via overlap PCR to replace *malE* amino acid residues 133–140, flanked at the *n*-terminus by the amino acids PDPGSGSG, and at the *c*-terminus by PDPGS. This extended linker confers higher protein expression, along with sufficient length at the *n*-terminus to preclude contributions from residues other than glycine or serine during MHC loading. The resulting chimeric proteins were named based on the numerical OVA sequence of the inserted peptide. For instance, a chimera containing all registers of the OVA peptide is termed *malE*_323–337. Non-wild type residues introduced into *malE_*327–337 are named with respect to register four positions, such that a residue change from valine to methionine at position P-2 is termed 327 V(-2)M. The modified *malE* genes were flanked with directional *SfiI* restriction sites and subcloned into pAK400 for periplasmic expression in *E. coli* (Krebber et al., 1997). The plasmid was sequenced to confirm epitope presence, transformed into strain BL21, grown overnight at 37**°**C in 1–4 L shake flasks. After replacing spent media with fresh terrific broth, protein expression was induced with 1 mM IPTG for 4–24 hours at 25 C. The cells were harvested by centrifugation and cell pellets were resuspended using ice-cold 0.1 M Tris pH 7.4-buffered 0.75 M sucrose at 3 mL per gram of cell pellet. An osmotic shock to release the periplasmic contents was performed by addition or 3 mL 1.0 mM EDTA per gram of cell pellet and approximately 0.3 mg/mL lysozyme followed by the addition of 6 mM MgCl_2_ on ice. After an additional centrifugation step, the supernatant was dialyzed against 10 mM Tris pH 7.4, 0.5 M NaCl with 12–14 kDa MWCO dialysis tubing. Chimeric proteins were recovered via immobilized metal affinity chromatography (IMAC) with the *c*-terminal His tag and size exclusion chromatography (Superdex 75; GE Healthcare) in HBS and stored at −80**°**C prior to use.

### Antigen Presenting and T Cell Hybridoma Cell Lines

The murine A20 B cell lymphoma line presenting I-A^d^ MHC was acquired from ATCC (TIB-208). An A20 variant line with a mutated, non-functional HLA-DMα gene, 3A5 [34], [35] was transferred from Dr. Kenneth Rock, Dana Farber Cancer Institute, Boston, Massachusetts. The murine DO11.10 T cell hybridoma line was transferred from Dr. Philippa Marrack, National Jewish Health Center, Denver, Co. The murine HEL11–25 responsive T cell hybridoma [25] was a generous gift from Dr. Andrea Sant, University of Rochester Medical Center, NY. All cells were maintained in DMEM supplemented with 10% FBS, 50 Units penicillin/50 mg streptomycin per mL and 2 mM L-glutamine.

### T Cell Activation Experiments

Each well of a 96-well plate was seeded with 15,000 A20 cells, 15,000 T cell hydridoma cells and peptide, *malE* chimera protein or control peptide/protein per well in DMEM media without L-glutamine, except where noted. After a 24-hour incubation at 37**°**C, 5% CO_2_, supernatant was transferred to a new plate and stored at −80**°**C prior to IL-2 quantification assays. All peptide/protein doses were performed in triplicate wells and all experiments replicated at least twice.

For *malE* chimeras and control protein experiments, the relevant protein was thawed, concentrated using 30K molecular weight cut-off centrifugal filter units (Millipore UFC903024) and filter-sterilized. Proteins were then quantified via absorbance at 280 nm using a predicted extinction coefficient and molecular weight, diluted to equal concentrations, the concentration confirmed and serially diluted in 96-well plates. In each well, the volume fraction of protein in HBS solution added did not exceed 10%, and an FBS concentration of 10% was maintained in all wells. Proteins were pre-incubated for four hours with antigen presenting cells (A20 or 3A5) cells prior to a 24-hour incubation with T cell hybridomas. The IL-2 concentration in the supernatant was quantified using a matched pair ELISA quantified with an IL-2 standard as described (BD #555148). All experiments were performed at least twice, and all wells were measured in triplicate. Data was analyzed with Graphpad Prism 5 (La Jolla, CA).

Glutamine was excluded from experiments (except where noted) to minimize cell growth, as preferential growth was observed in inverse proportion to T cell stimulation. Since overall IL-2 release is affected by T cell to B cell ratio, and assays were performed only at a limited number of discrete doses, we reasoned that differential growth could potentially mask small differences in the activity of specific peptides. The absence of glutamine lowered overall IL-2 release and required larger concentrations of peptide or protein to reach a given release.

### Predicted Peptide-MHC Docking

The coordinates for I-A^d^ and the OVA 323–334 peptide were extracted from PDB 1IAO. The ovalbumin peptide was extended from the c-terminus to residue 338 and manually extended such that backbone dihedral angles (φ, ψ) approximate those observed in an extended polyproline type II helix (−75, 150). Computational peptide docking was performed with ClusPro [36].

## Supporting Information

Figure S1 **Ovalbumin registers one and three are predicted as stably associated with I-A^d^ MHC by computational docking.** Peptide and MHC coordinates of 323–339 and I-A^d^ were extracted from PDB 1IAO and computationally docked using Cluspro (*Comeau et al*, 2004). Shown are peptide alignments of the register one wild type crystal structure (green) with the high-scoring ClusPro predicted register one alignment (blue), and ClusPro predicted register three alignment (yellow). Registers two and four were not predicted.
(TIF)
