# Supplementary figures and images for: Flanking Residues Are Central to DO11.10 T Cell Hybridoma Stimulation by Ovalbumin 323–339
Source: PLoS One. 2012 Oct 23;7(10):e47585. doi: 10.1371/journal.pone.0047585 (PMC3479146; doi:10.1371/journal.pone.0047585)

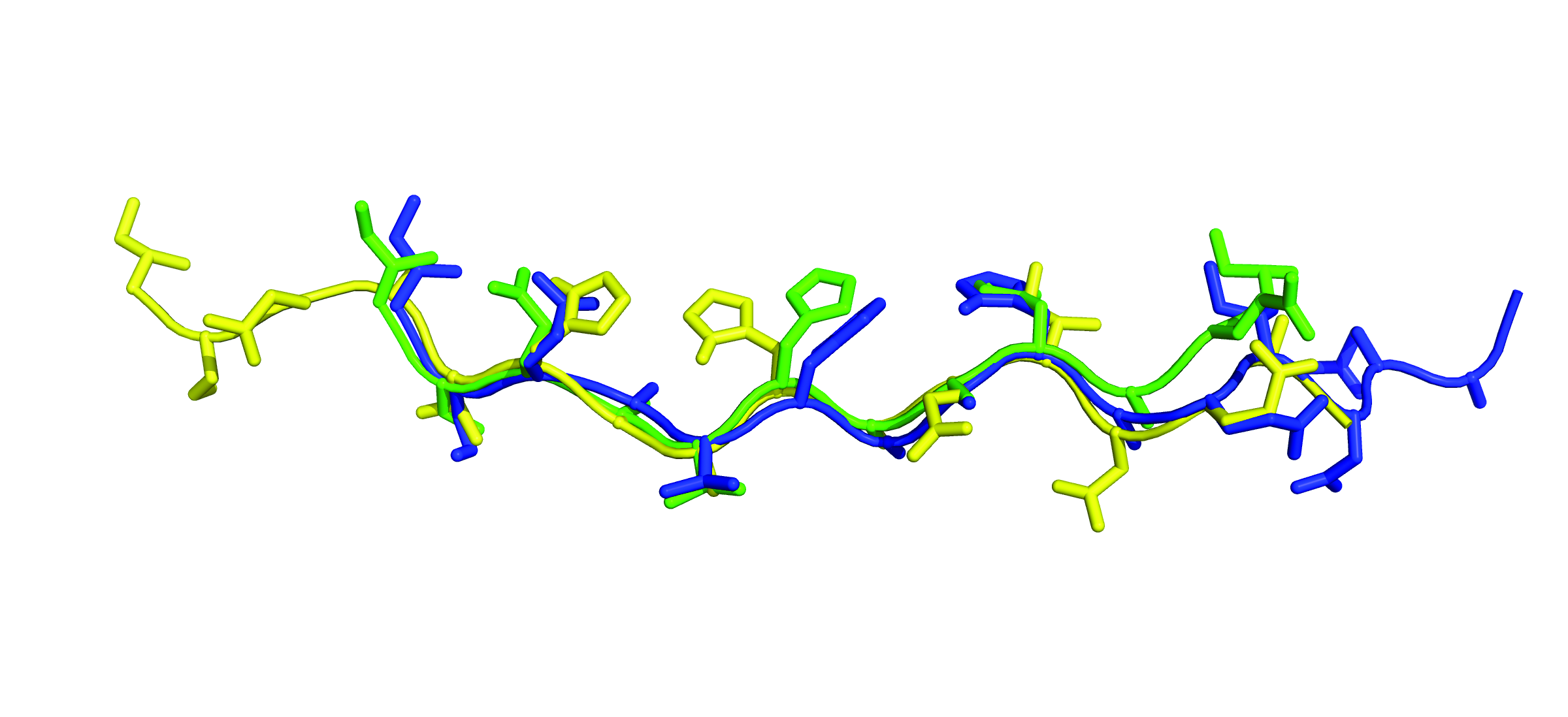

Supplement: Figure S1 — Ovalbumin registers one and three are predicted as stably associated with I-Ad MHC by computational docking. Peptide and MHC coordinates of 323–339 and I-Ad were extracted from PDB 1IAO and computationally docked using Cluspro (Comeau et al, 2004). Shown are peptide alignments of the register one wild type crystal structure (green) with the high-scoring ClusPro predicted register one alignment (blue), and ClusPro predicted register three alignment (yellow). Registers two and four were not predicted. (TIF) [file pone.0047585.s001.tif]
